# Biological Evaluation of Uridine Derivatives of 2-Deoxy Sugars as Potential Antiviral Compounds against Influenza A Virus

**DOI:** 10.3390/ijms18081700

**Published:** 2017-08-04

**Authors:** Ewelina Krol, Ilona Wandzik, Martyna Krejmer-Rabalska, Boguslaw Szewczyk

**Affiliations:** 1 Department of Recombinant Vaccines, Intercollegiate Faculty of Biotechnology of University of Gdansk and Medical University of Gdansk, Abrahama 58, 80-307 Gdansk, Poland; martyna.krejmer@biotech.ug.edu.pl (M.K.-R.); szewczyk@biotech.ug.gda.pl (B.S.); 2 Department of Organic Chemistry, Bioorganic Chemistry and Biotechnology, Faculty of Chemistry, Silesian University of Technology, Krzywoustego 8, 44-100 Gliwice, Poland; Ilona.Wandzik@polsl.pl

**Keywords:** influenza A virus, antiviral compounds, uridine, 2-deoxy sugars, glycosylation inhibition

## Abstract

Influenza virus infection is a major cause of morbidity and mortality worldwide. Due to the limited ability of currently available treatments, there is an urgent need for new anti-influenza drugs with broad spectrum protection. We have previously shown that two 2-deoxy sugar derivatives of uridine (designated IW3 and IW7) targeting the glycan processing steps during maturation of viral glycoproteins show good anti-influenza virus activity and may be a promising alternative approach for the development of new anti-influenza therapy. In this study, a number of IW3 and IW7 analogues with different structural modifications in 2-deoxy sugar or uridine parts were synthesized and evaluated for their ability to inhibit influenza A virus infection in vitro. Using the cytopathic effect (CPE) inhibition assay and viral plaque reduction assay in vitro, we showed that compounds **2**, **3**, and **4** exerted the most inhibitory effect on influenza virus A/ostrich/Denmark/725/96 (H5N2) infection in Madin-Darby canine kidney (MDCK) cells, with 50% inhibitory concentrations (*IC*_50_) for virus growth ranging from 82 to 100 (μM) without significant toxicity for the cells. The most active compound (**2**) showed activity of 82 μM with a selectivity index value of 5.27 against type A (H5N2) virus. Additionally, compound **2** reduced the formation of HA glycoprotein in a dose-dependent manner. Moreover, an analysis of physicochemical properties of studied compounds demonstrated a significant linear correlation between lipophilicity and antiviral activity. Therefore, inhibition of influenza A virus infection by conjugates of uridine and 2-deoxy sugars is a new promising approach for the development of new derivatives with anti-influenza activities.

## 1. Introduction

Influenza is a highly contagious acute viral infection with high morbidity and mortality rates in humans and animals worldwide [1]. About 20% of the world’s population is infected by the virus, which results in around 250,000–500,000 deaths annually [2]. The H1N1 and H3N2 influenza virus subtypes are the most prevalent and the main reason for several recent pandemics in humans, including the 2009 H1N1 pandemic with high morbidity. Vaccines and prophylaxis with antiviral drugs are currently two main strategies used to control influenza infections. Vaccination strategies are the best method of prevention. However, they have to be updated regularly to account for antigenic changes of the viral glycoproteins. Moreover, vaccines are only around 50% effective in the elderly, where most of the mortality occurs. Additionally, in the case of an epidemic or pandemic caused by a new strain, development of an effective vaccine requires from three to six months using the conventional egg-based method, which can be too slow to be effective against the spread of infection. Due to the lack of effectiveness of vaccines against rapidly emerging mutant viruses during seasonal epidemics and sporadic pandemics, antiviral drugs against influenza virus may represent the first line of defence, especially in the beginning of an epidemic until a suitable, effective vaccine becomes available.

Despite very intensive research for new antivirals, few compounds representing two classes of influenza virus inhibitors are currently available for clinical use: M2 ion-channel blockers (amantadine and rimantadine) [3,4] and neuraminidase (NA) inhibitors (oseltamivir and zanamivir) [5,6]. Other NA inhibitors, like Laninamivir and Peramivir, have been approved in North Asia but are still in clinical trials in Europe and North America [7]. M2 inhibitors that prevent viral uncoating are active only against influenza A viruses [8]. Although both amantadine and rimantadine show strong anti-influenza activity, the rapid appearance of drug resistance in circulating virus strains restricts the use of such drugs for influenza prevention and treatment [9,10,11]. By blocking the release of newly formed viral particles, neuraminidase inhibitors prevent the spread of the influenza A and B viruses [12]. Despite the fact that the target site for NA inhibitors is relatively well conserved, a number of resistant mutations in the NA gene have arisen rapidly as a consequence of drug induced selective pressure [13,14,15]. In the USA, the number of oseltamivir-resistant H1N1 viruses quickly increased from 0.5% in 2006–2007 to 99% in the 2008–2009 season [16,17]. In Europe and North America, oseltamivir-resistant H1N1 viruses were also detected and are now widely circulating [18,19,20]. Moreover, highly pathogenic avian influenza strains such as H7N9 and H5N1, with the potential to cause a new influenza pandemic, are also resistant to oseltamivir [21]. The limitations of current drug therapy and the increasing emergence of multiple drug-resistant influenza strains calls for a new generation of broad-spectrum anti-influenza drugs with an alternative mode of action that would be effective against a wide array of influenza strains.

Hemagglutinin (HA) and NA are membrane glycoproteins abundant on the viral surface. They play an important role in the influenza virus life cycle and constitute important targets for anti-influenza drug design. HA is involved in viral entry. It interacts with host cell receptors containing sialic acids and plays a role in membrane fusion [22]. NA catalyzes the removal of sialic acids and releases the progeny viruses from the complexes with host proteins at late stages of infection [23,24]. Hemagglutinin and neuraminidase are highly *N*-glycosylated proteins. HA and NA contain three to nine and four potential *N*-glycosylation sites, respectively [25,26], and their number and types depends on the virus subtype and strain [27,28]. The most conserved *N*-glycosylation occurs in the stalk region of the HA protein [29].

**Figure 1:**
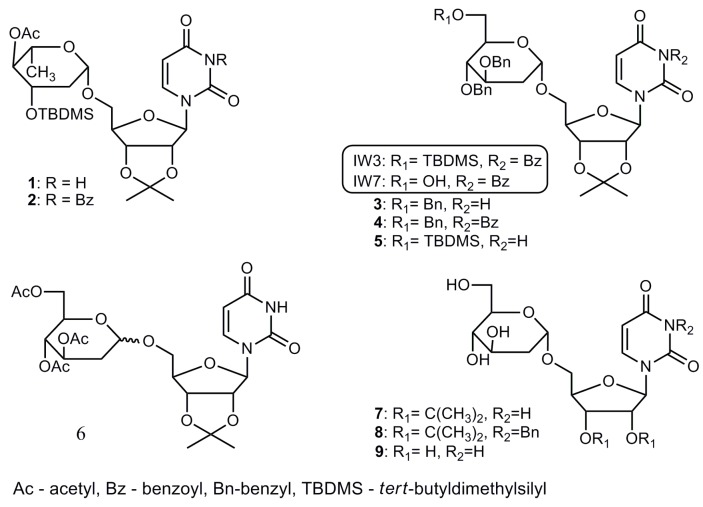
Structures of 2-deoxy sugar derivatives of uridine active against influenza A virus.

The composition of oligosaccharide chains in viral glycoproteins can be modulated by glycosyltransferases (GTs) during maturation. Alteration of glycosylation by effective inhibitors of GTs may affect viral survival. A number of natural products have been found to alter glycosylation, e.g., tunicamycin, which inhibits viral coat formation [30]. Recently, we have synthesized several compounds that were donor substrate analogues of GTs. These compounds can prevent glycosylation by occupying the active site of the enzyme [31,32,33]. They contain uridine moiety and mimic the uridine diphosphate (UDP)-sugar natural substrates. We have previously reported that two compounds from this series—2-deoxy sugar derivatives of uridine (IW3 and IW7) (Figure 1)—showed good anti-influenza virus activity in vitro with IC_50_ of 72 and 63 µM, respectively [34]. It was concluded that IW3 and IW7 block the influenza virus by targeting the glycan process during the synthesis of viral glycoproteins. We have shown that both compounds impaired maturation of influenza viral proteins by inhibiting the late step of *N*-glycosylation process, which differs from the mechanism of currently used anti-influenza drugs.

In this study, a number of IW3 and IW7 analogues featuring different structural modifications in 2-deoxy sugar or uridine parts were synthesized and further evaluated for their antiviral activity against influenza A virus. The new data show that some of the synthesized compounds possess good antiviral activities in vitro and that some modifications improved the selectivity indexes compared to the previously described IW7 compound.

## 2. Results

### 2.1. Chemistry

Recently, we reported that 2-deoxy sugar derivatives of uridine, compounds IW3 and IW7 (Figure 1), exhibited efficient inhibitory effects against H5N2 and H1N1 influenza A viruses in Madin-Darby canine kidney (MDCK) cells [34]. In the current work, we investigated the effect of various protecting groups on antiviral activity. The protections of hydroxyl groups in 2-deoxy sugar part, as well as hydroxyl groups in ribose part or uracil nitrogen, were applied. In a series of compounds, totally deprotected compounds were synthesized for comparison. Compounds **1**–**9** were synthesized in addition reactions of uridine acceptors to the double bond of glycals using the Falck-Mioskowski protocol [35]. The synthesis of compounds **5**, **7**, **8**, and **9** was published in our previous reports [32,33], while the synthesis and structural data of compounds **1**, **2**, **3**, **4**, and **6** is presented in Supplementary Material Sections S1 and S2.

### 2.2. Antiviral Activity of Newly Synthesized Compounds against Influenza A Virus

The cytotoxicity of synthesized compounds in MDCK cells was first evaluated by non-radioactive cell proliferation assay (MTS). For each compound, *CC*_50_ values, corresponding to a 50% cytotoxic effect after 48 h of inhibitor treatment, were determined (Table 1). There was no cytotoxicity observed in cells treated with the highest concentrations of DMSO (solvent) used in the screening.

**Figure 2:**
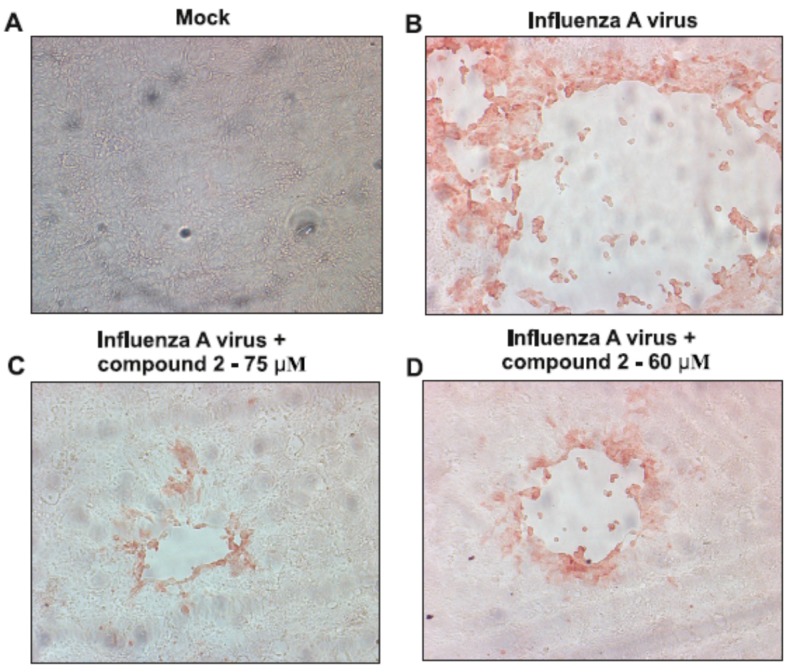
Reduction of influenza A virus-induced cytopathic effect by compound **2**. Madin-Darby canine kidney (MDCK) cells were not-infected (**A**) or infected with influenza virus A/ostrich/Denmark/725/96 (H5N2) (**B**–**D**). At 1 h p.i., the virus was removed and the cells were treated with 75 µM (**C**) or 60 µM (**D**) of compound **2** or left untreated (positive control—**B**). 24 h p.i., the inhibitory effect of compound **2** on virus replication was evaluated by immunostaining using a monoclonal anti-M1 antibody. Images under a Nicon Eclipse TE300 microscope (Nicon Corporation, Tokyo, Japan) at 100× magnification.

The antiviral activities of synthesized 2-deoxy sugar derivatives of uridine **1**–**9** against influenza virus A/ostrich/Denmark/725/96 (H5N2) were evaluated using cytopathic effect (CPE) inhibition assay and plaque reduction assay. Influenza A/ostrich/Denmark/725/96 virus causes a severe CPE in MDCK-infected cells including cell rounding, detachment and death. A reduction in influenza A virus-induced cytopathic effect after 24 h incubation indicated antiviral activity of the tested compounds. The initial results showed that all nine synthesized compounds exhibit low to high antiviral activity against influenza A virus. It was found that compounds **2**, **3**, and **4** significantly reduced the CPE caused by infection in MDCK cells, while the remaining compounds were less active. The CPE was reduced when appropriate doses of each compound were added to MDCK-infected cells, which suggests that all compounds were able to protect cells from virus-induced cell death. The example of the effect of compound **2** on CPE reduction is shown in Figure 2.

A plaque reduction assay was carried out to confirm the efficacy of tested compounds on influenza virus propagation and to measure the accurate *IC*_50_ values. MDCK cells were infected with H5N2 influenza strain (*MOI* = 0.01) and incubated with overlay medium containing various concentrations of compounds **1**–**9**. After three days, cells were immunostained with specific monoclonal anti-M1 antibody to measure the extent of viral infection. Oseltamivir carboxylate, an inhibitor of influenza neuraminidase, was used as a positive control. This compound displayed an *IC*_50_ value of about 0.15 μM and low cytotoxicity (*CC*_50_ > 100 μM) in our assay.

**Figure 3:**
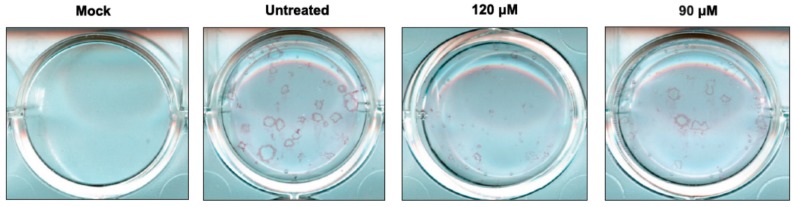
Dose-dependent reduction of influenza A viral plaque formation after treatment with compound **2**. MDCK cells were infected with influenza virus A/ostrich/Denmark/725/96 (H5N2) or mock infected. At 1 h p.i., the virus was removed and the cells were incubated with overlay medium containing compound **2**. 3 days p.i., cells were fixed and influenza A virus plaques were detected by immunostaining with specific, monoclonal anti-M1 antibody.

The *IC*_50_ values for the most active compounds **2**, **3**, and **4** were in range of 82–100 μM (Table 1). One of the least polar compound **2** exhibited the highest activity. The average size and plaque number in compound **2**–treated cells were markedly reduced in a dose-dependent manner (Figure 3).

The analysis showed that the presence of benzoyl group in the uracil part in compound **2** significantly improved antiviral effect (*IC*_50_ = 82 μM) and reduced the cytotoxicity in comparison to compound **1** (*IC*_50_ = 175 μM). This is in contrast to 2-deoxy glucose series where no significant increase of activity was observed for compound **4** (*IC*_50_ = 99 μM) in comparison with compound **3** (*IC*_50_ = 100 μM). Different protection of hydroxyl groups in 2-deoxy-glucose part did not affect the activity when nonpolar protective groups were introduced. The antiviral activity was slightly reduced compared to compound **2**. Significant loss of activity was observed for compound **6** (*IC*_50_ = 575 μM) containing relatively polar per-O-acetylated 2-deoxy-glucose part. Moreover, the similar effect of reduced activity was noticed for compounds **7**–**9** containing totally deprotected hydroxyl groups in 2-deoxy-glucose part (*IC*_50_ values of 721, 346 and 410 μM, respectively), although the cytotoxicity of these compounds was low. It seems that lipophilicity of studied compounds might be a key parameter influencing the viral activity.

### 2.3. Quantitative Structure-Activity Relationship Analysis between Antiviral Activity and Lipophilicity

Molecular lipophilicity is a key factor in the description of pharmacokinetic properties of a drug and its interaction with molecular targets. This physicochemical descriptor characterizes the affinity of a drug for lipophilic environment. It is usually defined as the partition coefficient (P) between n-octanol and water. In most cases this value is presented as log*P*. The experimental logP by direct shake-flask method can not always be determined for some compounds due to technical reasons. Instead, calculated log*P* or other experimental lipophilicity parameters could be determined. Theoretical log*P* values for the target compounds can be calculated using different algorithms. Recently, we have showed a good agreement between experimental data and theoretical log*P* values for twenty one uridine derivatives [36]. A good agreement was obtained when XLogP3 algorithm [37] was applied, thus in this study XlogP3 have been calculated for structures **1**–**9**.

As an alternative to calculated logP, different experimental lipophilicity parameters, e.g., the chromatographic lipophilicity index (*R*_Mw_) might be used. It was reported that there is a strong linear relationship between *R*_Mw_ parameter and experimental log*P* for a certain family of chemically related compounds [38]. Experimental lipophilicity parameters *R*_Mw_ of studied compounds were determined by reverse phase thin layer chromatography (RP-TLC) according to the method previously described [36] (Supplementary Material Section S3). It was possible to determine *R*_Mw_’s for all compounds, except for compound **9** because of very low lipophilicity. In this work, experimentally obtained *R*_Mw_, and computed XlogP3 data (Table 2) were analyzed independently in order to find relationships between lipophilicity and antiviral activity against influenza A virus expressed as *p*[*IC*_50_].

A good linear correlation between the antiviral activity of the compounds **1**–**9** and their lipophilicity was observed. More hydrophobic compounds, characterized by higher *R*_Mw_ or *X*log*P3* values had increased antiviral activity, which can be expressed by Equations (1) and (2). Better fit was observed between activity and experimental *R*_Mw_ parameter (*R* = 0.96) in comparison with calculated *X*log*P3* (*R* = 0.92). The fitness of the model was checked by the coefficient of determination (R2) and standard error of the regression (se). All statistical parameters are described in methods.

*p*[*IC*_50_] = 3.091 (±0.080) + 0.126 (±0.014) × *R*_Mw_ (1)

*n* = 8, *R* = 0.96, *R*^2^ = 0.93 *s_e_* = 0.11, F (1,6) = 71.6, *p* = 0.0001

*p*[*IC*_50_] = 3.505 (±0.057) + 0.112 (±0.017) × *X*Log*P3* (2)

*n* = 9, *R* = 0.92, *R*^2^ = 0.85 *s_e_* = 0.15, F (1,7) = 41.3, *p* = 0.0003

It was found that compounds with high antiviral activity have to contain hydrophobic fragments, e.g., benzyl, benzoyl, *tert-*butyldimethylsilyl or isopropylidene groups. The most active compounds from the series were the most lipophilic compounds **2**–**4**, conversely the least active compounds were the most polar derivatives **7**–**9** containing totally deprotected 2-deoxy sugar units.

### 2.4. Inhibitory Effects of Compound **2** on Different Stages of Viral Replication Cycle

**Figure 4:**
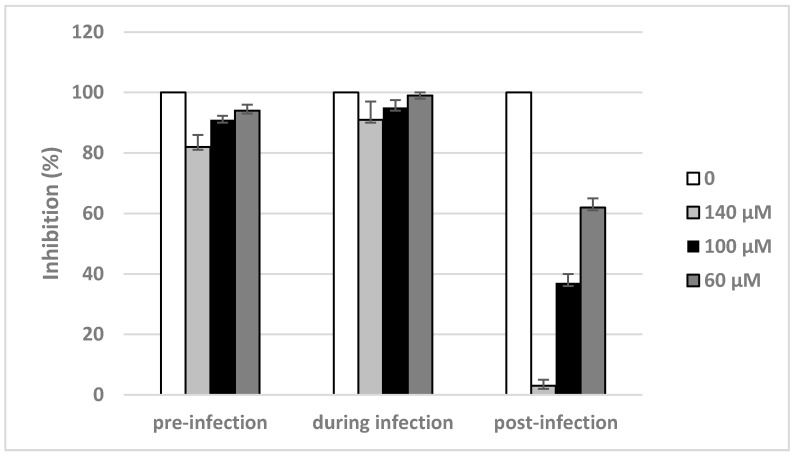
Inhibitory effects of compound **2** on different stages of viral infection. Compound **2** was added to MDCK cells overnight before adsorption (I), during viral adsorption for 1 h (II) or post-viral adsorption for 72 h (III). A/ostrich/Denmark/725/96 (H5N2) infected MDCK cultures were incubated for 72 h. The cell viability was evaluated by MTS assay.

In the light of promising activity of compound **2**, the activity of this inhibitor was more thoroughly examined. To determine the stages by which compound **2** acts during the influenza A virus life cycle, a time-of-addition studies were performed according to the scheme: overnight before virus adsorption (I), during virus adsorption for 1 h (II) or post-virus adsorption for 72 h (III). After incubation in case of I and II, the inhibitor was removed and cells were grown for 72 h in fresh medium. The analysis showed that compound **2** inhibited the propagation of influenza virus only when it is added to the cells after viral infection (Figure 4). The obtained results strongly suggested that this compound target post-adsorption steps of influenza A virus replication cycle.

### 2.5. Inhibitory Effect of Compound **2** on Influenza A Protein Synthesis

**Figure 5:**
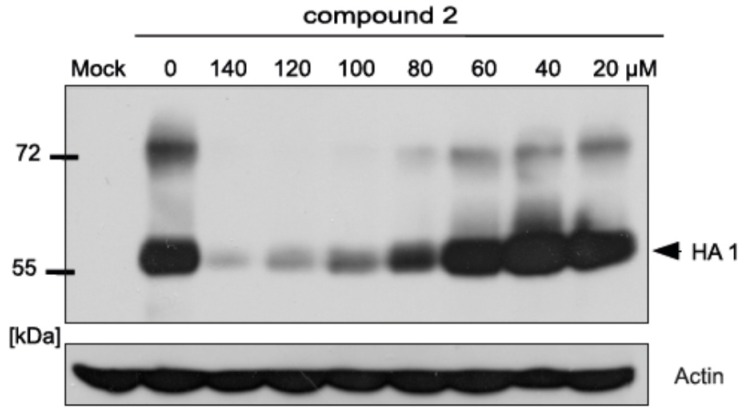
Effect of compound **2** on influenza A virus HA glycoprotein synthesis. Madin-Darby canine kidney cells (MDCK) cells were infected with influenza A virus (pandemic human A/H1N1 virus) and treated with various concentrations of compound **2**. At 48 h post-infection, cells were lysed, and proteins were separated by SDS–PAGE (10% polyacrylamide) under reducing conditions. Western blot analysis was performed using the specific rabbit polyclonal serum anti-sHA and anti-β-actin monoclonal antibodies. HA: Hemagglutinin.

We have previously shown that two synthesized by us 2-deoxy sugar derivatives of uridine (compounds IW3 and IW7), belonging to *N*-glycosylation inhibitors, effectively blocked influenza A virus propagation by affecting the synthesis of viral glycoproteins [34]. Due to the fact that the new compounds were synthesized as derivatives of IW3 and IW7 compounds, we wanted to check the effects of further modifications of these compounds on viral glycoprotein synthesis. Compound **2** was found to be the most active in the series, therefore this compound was selected for further investigation. To explore the inhibitory effect on the replication of influenza A virus, the influence of compound **2** on protein synthesis was determined. Western blotting analysis showed that the yield of HA protein in cells infected with pandemic human influenza A/H1N1 virus was reduced by compound **2** in a dose-dependent manner (Figure 5). These results were in agreement with the results obtained previously for IW3 and IW7 compounds. Furthermore, with the highest doses of compound **2**, the mature form of HA protein were not detected. Moreover, we were not able to detect the unglycosylated or underglycosylated forms of HA protein, probably due to the fact that incorrectly matured proteins are rapidly degraded. The consistent expression levels of β-actin indicated that the tested doses of compound **2** did not affect the levels of host cellular proteins.

## 3. Discussion

Influenza is an acute respiratory illness with high morbidity and mortality globally. Some circulating influenza subtypes can cause massive global epidemics, causing panic in human population. Anti-influenza treatment with current antivirals often leads to drug resistance in circulating influenza strains, and this fact calls for new drugs targeting alternative steps of viral development. Numerous herbs and their bioactive ingredients started to play role as an important source of therapeutic agents in recent years [39,40]. Even though some of them showed good antiviral activity in vitro, most of their antiviral activity is still associated with the influenza viral replication process. However, there are some reports demonstrating the antiviral activity of natural compounds via interaction with viral structural proteins like hemagglutinin [41,42].

Hemagglutinin (HA) and neuraminidase (NA) are enveloped proteins of the influenza virus that mediate viral entry and release. HA interacts with host cell receptors containing sialic acids facilitating virus entry and plays a role in membrane fusion [22]. NA is a sialidase catalyzing the removal of sialic acids from the complexes between progeny virus and host cell components which allows for the release of newly formed viruses [23]. The roles of these proteins in the influenza virus life cycle make them prospective candidates for novel antiviral strategies. Both NA and HA are glycoproteins that contain 4 and 3–9 N-linked glycan chains, respectively [25,26].

*N*-glycosylation is one of the most important steps in maturation of viral proteins. It is essential for the stability and correct folding of glycoproteins and it also strongly influences other important biological functions. It has been found that the arrest of glycosylation of viral glycoproteins by different natural or chemically synthesized inhibitors usually results in antiviral effects [43,44,45,46]. The antibiotic tunicamycin is one of the best known inhibitors of *N*-glycosylation process with significant antiviral activity. However, its high toxicity in vivo prevents the use of this antibiotic as a therapeutic agent [47,48].

This significant antiviral activity of tunicamycin encouraged us to synthesize compounds mimicking the tunicamycin structure and to check their antiviral activity. In our previous studies, we have demonstrated that two compounds belonging to tunicamycin derivatives—uridine derivatives of 2-deoxy sugars (named IW3 and IW7)—possess significant antiviral activity against classical swine fever virus [49]. We have shown that these two compounds affect protein glycosylation similarly to tunicamycin, but they are significantly less toxic. Moreover, our other previous report consistently confirmed that both IW3 and IW7 compounds, due to the universal mechanism of action, were also active against influenza A virus H5N2 and H1N1 strains at non-toxic concentrations targeting the maturation of viral proteins [34]. This unique effect of both compounds on the HA protein prompted our further studies focused on the examination of the effect of a wide array of other synthesized compounds on the yield of influenza virus.

In the current study, nine novel 2-deoxy sugar derivatives of uridine were synthesized and screened for their antiviral activity against influenza A virus. We showed that compounds **2**, **3**, and **4** exerted the most significant inhibitory effect on in vitro influenza virus infection in the series evaluated in CPE inhibition assay as well as in the plaque reduction assay in MDCK cells using A/ostrich/Denmark/725/96 (H5N2) virus. Our analysis showed that the antiviral activity of tested compounds is enhanced by the addition of hydrophobic fragments, e.g., benzyl, benzoyl, tert-butyldimethylsilyl, or isopropylidene groups. The data showed that some modifications improved the selectivity indexes of new synthesized compounds compared to the previously published IW7 compound (Table 1). Linear correlation of lipophilicity and antiviral activity was observed: the more hydrophobic substituents increased antiviral activity (Equations (1) and (2)). It might be hypothesized that the antiviral activity is not specific, and only the highly lipophilic compounds could cross cell membranes; thus, the activity is related to pharmacokinetic aspects. Alternatively, hydrophobic interactions between the highly lipophilic compounds and receptors might occur. One of the most lipophilic, compound **2**, with the presence of benzoyl group in the uracil part, showed the highest antiviral activity against H5N2 influenza A virus strain among all tested compounds, with an *IC*_50_ value of 82 µM and low cytotoxicity (*CC*_50_ of 432 µM) as observed in plaque reduction assay (Figure 3). Our studies have confirmed that the mechanism of the antiviral activity of compound **2** against influenza virus may be the same as was shown for IW3 and IW7 compounds. Greater activity of compound **2** was observed when this inhibitor was added after viral adsorption, which suggests that it acts predominantly on the intracellular steps of the viral replication cycle (Figure 4). Moreover, we have examined the effect of this new lead compound on the synthesis of viral proteins. These studies indicated that the reduction in influenza virus production (Figure 2 and Figure 3) can be associated with the inhibition of viral glycoprotein synthesis in infected cells. We have demonstrated that compound **2** caused a dose-dependent decrease of HA glycoprotein production (Figure 5). However, the underglycosylated forms of HA were not detected, suggesting that such incorrectly matured polypeptides are degraded very quickly in host cells.

The role of *N*-glycosylation process in the life cycle of influenza virus has been shown in many reports [50,51,52,53,54,55]. For both HA and NA proteins, the number and location of glycans on the polypeptide chain are crucial for biological activity of these proteins. The importance of tip glycans of HA was reported by Wagner and coworkers [54]. It was found that the lack of one or two glycans led to the significant arrest of the growth of HA mutants in cell culture. Also, as shown by Saito and Yamaguchi, the transport of NA to the host cell surface is impaired by the inhibition of *N*-glycosylation resulting in the restrictions on the release of viral progeny [53]. In light of the presented data, the new studies in the search for inhibitors targeting viral glycoprotein maturation are highly justified.

In summary, the presented data showed that 2-deoxy sugar derivatives of uridine may constitute a novel class of inhibitors with mechanism of action different from currently used anti-influenza drugs. The study demonstrated that some of synthesized compounds demonstrate good antiviral activities in vitro and that some modifications improved the selectivity indexes compared to the previously published IW7 compound. Overall, our data suggest that further modification of the compound **2** could result in hit compounds endowed with potentially increased antiviral activity.

## 4. Materials and Methods

### 4.1. Cells, Viruses and Antiviral Compounds

Madin-Darby canine kidney cells (MDCK; ATCC^®^ CCL-34™) were grown in Dulbecco’s Modified Eagle’s Medium (D-MEM) (Sigma–Aldrich, St. Louis, MI, USA), containing 10% heat-inactivated fetal bovine serum (FBS), 2 mM l-glutamine, 0.2% bovine serum albumin, 25 mM HEPES buffer, 100 U/mL penicillin, 100 µg/mL streptomycin, at 37 °C under 5% CO_2_.

The avian influenza virus A/ostrich/Denmark/725/96 (H5N2) was obtained from the Department of Poultry Diseases, National Veterinary Research Institute, Pulawy, Poland. Isolate 32U of the pandemic human influenza A/H1N1 virus) from the collection of the Department of Recombinant Vaccines, University of Gdansk, Poland, was also used in this study. Influenza A viruses were grown in MDCK cells in the presence of 2 µg/mL TPCK (l-1-Tosylamide-2-phenylethyl chloromethyl ketone)—trypsin (Sigma–Aldrich, St. Louis, MI, USA). Virus titers were determined by plaque assay.

The stocks solutions of synthesized compounds were prepared by dissolving the reagents in dimethyl sulfoxide (DMSO) and stored in −20 °C until future use. Oseltamivir carboxylate was purchased from Santa Cruz Biotechnology (Heidelberg, Germany).

### 4.2. Cell Viability Assay

MDCK cell viability was determined by CellTiter 96 AQ_ueous_ non-radioactive cell proliferation assay (MTS) (Promega: Madison, WI USA) as described previously [34]. Stock solutions of synthesized compounds were prepared by dissolving in dimethyl sulfoxide (DMSO) and stored at −20 °C. The half-maximal cytotoxic concentration (*CC*_50_) was calculated as the compound concentration required to reduce cell viability by 50%.

### 4.3. Cytopathic Effect (CPE) Inhibition Assay

Antiviral activity was evaluated in cytopathic effect (CPE) inhibition assay and plaque reduction assay by methods reported previously [34]. Briefly, in the CPE inhibition assay, confluent monolayers of MDCK cells, seeded in 6-well plates, were infected with influenza virus A/ostrich/Denmark/725/96 (H5N2) for 1 h at 37 °C. The virus was removed by washing with serum-free medium, and cells were overlaid with fresh serum-free medium supplemented with 2 µg/mL TPCK-trypsin and various concentrations of inhibitors. After 24 h, CPE was determined both by MTS assay and by immunostaining with a monoclonal anti-M1 antibody as described in plaque reduction assay.

### 4.4. Plaque Reduction Assay

Confluent monolayers of MDCK cells in 12-well plates were inoculated with influenza A virus for 1 h at 37 °C. After removal of the virus, the cells were washed with serum-free medium and overlaid with fresh serum-free medium supplemented with 1.2% Avicel (FMC BioPolymer, Philadelphia, PA, USA), 2 µg/mL TPCK-trypsin, and inhibitors at different concentrations. Three days post-infection, cells were washed with phosphate-buffered saline (PBS), fixed with 4% paraformaldehyde, and virus plaques were detected by immunostaining with anti-M1 antibody (diluted 1:1000 in PBS, 1% Tween 20, 5% FBS) followed by anti-mouse horseradish peroxidase (HRP)-conjugated secondary antibody (diluted 1:1000 in PBS containing 1% Tween 20 and 5% FBS). Plaques were visualized using the Vector Nova-Red kit (Vector Laboratories Ltd., Peterborough, UK) and counted. The 50% inhibitory concentration (*IC*_50_) was determined as the concentration at which the plaques area is reduced by 50% compared to untreated infected control. This value was calculated in both virus-induced CPE by MTS assay and immunohistochemical method in plaque reduction assay.

### 4.5. Time-of-Addition Studies

To examine the anti-influenza activity of compound **2** at different stages of the influenza life cycle, compound **2** was added overnight before virus adsorption (I), during viral adsorption for 1 h (II) and post-viral adsorption for 72 h. After incubation in case of I and II, the inhibitor was removed and the cells were grown for 72 h in fresh medium. Cell viability was determined using MTS assay.

### 4.6. SDS-PAGE and Western Blot Analysis

MDCK cells in 12-wells plates were infected with influenza A virus (*MOI* = 1) for 1 h at 37 °C. The inoculum was removed and the cells were washed with serum-free medium. Fresh serum-free medium containing 2 µg/mL TPCK-trypsin and inhibitors at various concentrations was added for 48 h. Cells were lysed at 4 °C for 1 h with TNET buffer (20 mM Tris–HCl (pH 7.4), 150 mM NaCl, 1 mM EDTA, 1% Triton X-100). Proteins were separated by SDS–PAGE under reducing conditions, transferred to PVDF membranes and detected with primary rabbit polyclonal serum anti-sHA (1:500 dilution) or anti-β-actin antibody (1:1000 dilution) followed by anti-rabbit or anti-mouse peroxidase (HRP)-conjugated secondary antibodies (diluted 1:2000). Antigen-antibody complexes were detected using Super Signal West Pico Substrate system (Pierce) on the X-ray films (Fuji, Tokyo, Japan).

### 4.7. Determination of Lipophilicity Parameters

Determination of *R*_Mw_’s of compounds **1**–**8** by RP-TLC was performed using procedures which have been described elsewhere [37] and is presented in Supplementary Material Section S3.

Theoretical partition coefficients XlogP3 of the examined compounds **1**–**9** were calculated using ALOGPS (version 2.1) software [38].

### 4.8. Quantitative Structure-Activity Relationship Analysis

Quantitative structure-activity relationship analysis was performed by linear regression analysis with Statistica (version 9.1). Values for coefficients in Equations (1) and (2) were found in the least square approach. The fitness of the model was checked by the coefficient of determination (*R*^2^) and standard error of the regression (*s_e_*). The statistical significance of the estimated parameters was checked by *F*-test and the corresponding probability value (*p*). Values *R*, *R*^2^, F, *s_e_*, and *p* are listed below Equations (1) and (2).

## Figures and Tables

**Table 1:** Cytotoxicity and antiviral activity of synthesized compounds against influenza A virus.

Compound	MW (g/mol)	*CC*_50_ (μM) ^a^	*IC*_50_ (μM) ^b^	SI ^c^
**1**	570	193	175 ± 8.22	1.10
**2**	674	432	82 ± 6.81	5.27
**3**	700	407	100 ± 9.84	4.07
**4**	804	480	99 ± 5.23	4.85
**5**	724	150	96 ± 7.64	1.56
**6**	556	620	575 ± 17.18	1.08
**7**	430	828	721 ± 23.21	1.15
**8**	520	669	346 ± 10.53	1.93
**9**	390	800	410 ± 11.22	1.95
IW3	828	640	72 ± 5.20	8.89
IW7	714	123	63 ± 4.17	1.95

^a^ Compound concentration required to reduce cell viability by 50%; ^b^ Compound concentration required to inhibit virus plaque production by 50%; Data are mean values ± S.D. from three independent experiments; ^c^ Selectivity index calculated as ratio of *CC*_50_ to *IC*_50_.

**Table 2:** Antiviral activity and lipophilicity parameters of 2-deoxy sugar derivatives of uridine.

Compound	*p*[*IC*_50_]	*R*_Mw_	*X*log*P3*
**1**	3.76	5.83	2.04
**2**	4.09	6.74	3.66
**3**	4.00	6.40	3.63
**4**	4.00	7.58	5.30
**5**	4.01	8.06	4.94
**6**	3.24	2.32	−0.74
**7**	3.14	0.19	−1.91
**8**	3.46	2.09	−0.24
**9**	3.39	-	−3.15

*p*[*IC*_50_] = −log[*IC*_50_], where IC_50_ is expressed in mole/L; *R*_Mw_—The chromatographic lipophilicity index determined experimentally by RP TLC; *X*log*P3*—Calculated partition coefficient.

## Supplementary Materials

Supplementary materials can be found at www.mdpi.com/1422-0067/18/8/1700/s1, references [56,57,58,59,60,61,62] are cited in the supplementary materials.

## Abbreviations

*CC*_50_	Compound concentration required to reduce cell viability by 50%
CPE	Cytopathic effect
HA	Hemagglutinin
IC_50_	Compound concentration required to inhibit virus plaque production by 50%
MDCK	Madin-Darby canine kidney cells
NA	Neuraminidase
S.D.	Standard deviations
SI	Selectivity index
TPCK	l-1-Tosylamide-2-phenylethyl chloromethyl ketone
