# Supplementary material for: Biological Evaluation of Uridine Derivatives of 2-Deoxy Sugars as Potential Antiviral Compounds against Influenza A Virus
Source: Int J Mol Sci. 2017 Aug 4;18(8):1700. doi: 10.3390/ijms18081700 (PMC5578090; doi:10.3390/ijms18081700)
Supplement: Supplementary file 1 [file ijms-18-01700-s001.pdf]

SUPPLEMENTARY MATERIAL

Biological Evaluation of Uridine Derivatives of 2-Deoxy Sugars as Potential Antiviral Compounds Against Influenza A Virus

Ewelina Krol [1,*], Ilona Wandzik [2], Martyna Krejmer-Rabalska [1] and Boguslaw Szewczyk [1]

[1] Department of Recombinant Vaccines, Intercollegiate Faculty of Biotechnology of University of Gdansk and Medical University of Gdansk, Abrahama 58, 80-307 Gdansk, Poland; ewelina@biotech.ug.gda.pl (E.K.); martyna.krejmer@biotech.ug.edu.pl (M.K.-R.); szewczyk@biotech.ug.gda.pl (B.S.)
[2] Department of Organic Chemistry, Bioorganic Chemistry and Biotechnology, Faculty of Chemistry, Silesian University of Technology, Krzywoustego 8, 44-100 Gliwice, Poland; Ilona.Wandzik@polsl.pl
* Correspondence: ewelina@biotech.ug.gda.pl; Tel.: +48-58-523-63-83

1. Chemistry—Results

Compounds **1-9** were synthesized in addition reactions of uridine acceptors to the double bond of glycals using the Falck-Mioskowski protocol (**Table S1, Scheme SI**) [35]. When D-glucal substrates **11** and **12** were used, adducts **5,** and **7-9** were synthesised as pure α-anomers. In contrast, the addition of uridine derivative **14** and **15** to L-rhamnal derivative **10** led to adduct **1** and **2** which were contaminated with β-isomer (α:β = 5:1). The worst stereoselectivity (α:β = 3.5:1) was observed when 3,4,6-tri-*O*-acetyl-D-glucal (**13)** reacted with 2,3-isopropylideneuridine (**14**). The structure of all synthesized compounds was unambiguously assigned on the basis of analytical and ^{1}H and ^{13}C NMR spectroscopy data. The ratio of stereoisomers were determined by examination of the ^{1}H NMR spectra of isomeric mixture. The equatorially oriented H-1'α proton of the major α isomer gave the characteristic broad doublet at δ 4.9 ppm with $J_{1,2ax}$ ca 2.5 Hz, while the axially oriented H-1'β proton was observed at δ 4.5 ppm as doublet of doublets with $J_{1,2eq}$ ca 1.9 Hz and $J_{1,2ax}$ 9.8 Hz.

Table S1. Synthetic details of 2-deoxy sugar derivatives of uridine.

Entry	Glycal substrate	Uridine substrate	Deprotection procedure	Product (α:β)
1	**10**	**14**	-	**1** (4:1)
2	**10**	**15**	-	**2** (5:1)
3	**11**	**14**	-	**3** (5:1)
4	**11**	**15**	-	**4** (4:1)
5	**12**	**14**	-	**5** (α)
6	**13**	**14**	-	**6** (3.5:1)
7	**11**	**14**	$Pd(OH)_2/C$, cyclohexene	**7** (α)
8	**11**	**16**	$Pd(OH)_2/C$, cyclohexene	**8** (α)
9	**11**	**17**	$Pd(OH)_2/C$, cyclohexene	**9** (α)

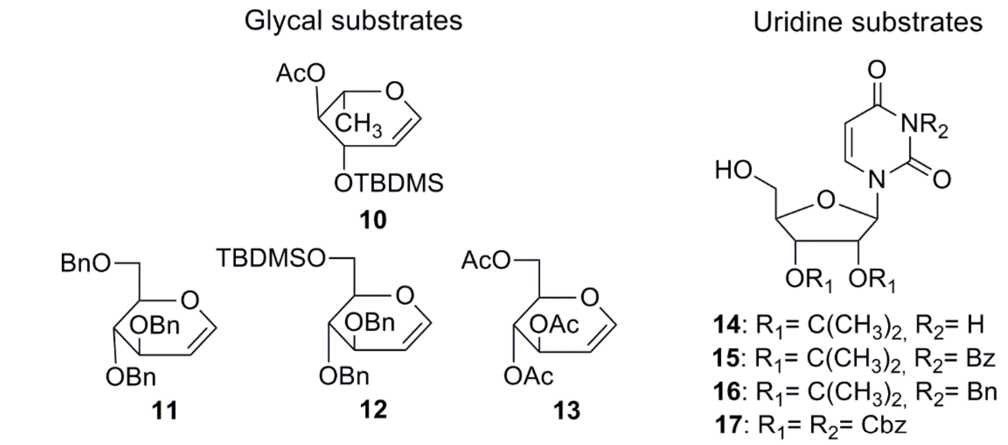

Ac - acetyl, Bz - benzoyl, Bn-benzyl, Cbz - benzyloxycarbonyl, TBDMS - *tert*-butyldimethylsilyl

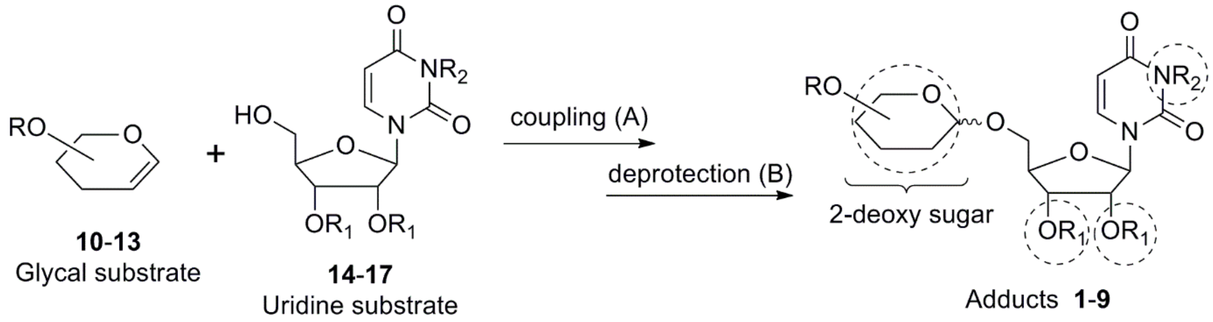

Scheme SI. Synthesis of compounds **1**–**9**. Reagents and conditions: A: TPHB, CH_2Cl_2, rt; B: $Pd(OH)_2/C$, cyclohexene, EtOH/THF, reflux

2. *Chemistry—Experimental Part*

Optical rotations were measured with a JASCO P-2000 polarimeter (Jasco Inc., Easton, MD, USA) using a sodium lamp (589 nm) at room temperature. NMR spectra were recorded with a Varian spectrometer (Varian, Inc., Palo Alto, CA, USA) (at a frequency of 300 MHz with TMS as internal standard. Mass spectra were recorded with a WATERS LCT Premier XE system (high resolution mass spectrometer with TOF analyzer) using electrospray-ionization (ESI) technique (Waters Corporation, Milford, MA, USA). Reactions were monitored by TLC on precoated plates of silica gel 60 F_{254} (Merck & Co. Inc., Kenilworth, NJ, USA) and visualized using UV light (254 nm). Column chromatography was performed on silica gel 60 (70-230 mesh, Fluka). Hexane/EtOAc or $CHCl_3$/MeOH were used as solvent systems. All evaporation were performed under reduced pressure at 50 °C.

Uridine substrates: 2',3'-O-isopropylidene-uridine (**14**) [56], 3-N-benzoyl-2',3'-isopropylidene-uridine (**15**) [57], 3-*N*-benzyl-2',3'-isopropylidene-uridine (**16**) [58] and N^3,2',3'-*O*-tris-(benzyloxycarbonyl)uridine (**17**) [33] were prepared according to the published procedures. Glycal substrates: 4-*O*-acetyl-3-*O*-*tert*-butyldimethylsilyl-L-rhamnal (**10**) [59], 3,4,6-tri-*O*-benzyl-D-glucal (**11**) [60], 3,4-di-*O*-benzyl-6-*O*-*tert*-butyldimethylsilyl-D-glucal (**12**) [61] and 3,4,6-tri-*O*-acetyl-D-glucal (**13**) [62] were prepared according to the published procedures. Chemicals were purchased from Aldrich and Fluka Chemical Companies and were used without purification. Solvents were dried and stored over molecular sieves (4Å) under an inert atmosphere.

2.1. General synthetic procedures

Procedure A. To a solution of glycal **10**, **11** or **13** (0.30 mmol) and uridine derivative **14**, **15** or **16** (0.30 mmol) in dry CH_2Cl_2 or acetonitril (5 mL) a catalytic amount of TPHB (10 mg, 0.03 mmol) was added. The mixture was kept at room temperature for 1-24 h. Then the reaction mixture was concentrated to give crude product purified directly by column chromatography with hexane/AcOEt 2:1 solvent system to yield adducts **1**, **2**, **3**, **4**, **6** as foamy solids. Compounds **5**, **7, 8** and **9** were synthesized and identified in our recent reports [31-33].

2.1.1. 4-O-Acetyl-3-O-tert-butyldimethylsilyl-2,6-dideoxy-L-arabino-hexopyranosyl-(1→5)-2',3'-isopropylideneuridine (1)

Glycal **10** (86 mg, 0.30 mmol) and uridine derivative **14** (85 mg, 0.30 mmol) in acetonitrile were subjected to general procedure A (reaction time 1 h) yielding adduct **1** as an inseparable α,β-anomeric mixture (120 mg, 70% yield, colourless foam).
^{1}H NMR ($CDCl_3$): (α:β = 4:1) δ 8.92, 8.81 (2 s, NH), 7.76, 7.39 (2 d, *J* 8.1 Hz, H-6), 5.95 (d, *J* 3.0 Hz, H-1'β), 5.81 (d, *J* 2.9 Hz, H-1'α), 5.76 (d, 1H, *J* 8.1 Hz, H-5α), 5.65 (d, 1H, *J* 8.1 Hz, H-5β), 4.88 (bd, *J* 2.9 Hz, H-1"α), 4.82 (dd, *J* 2.4, 6.1 Hz, H-2'), 4.78 (dd, *J* 2.9, 6.1 Hz, H-3'), 4.63 (t, *J* 9.2 Hz, H-4"), 4.50 (dd, *J* 1.9, 9.8 Hz, H-1"β), 4.42-4.30 (m, H-4'), 4.08 (m, H-3'β), 3.96 (ddd, *J* 5.3, 9.2, 11.2, H-3"α), 3.92 (dd, *J* 2.7, 9.3 Hz, H-5'b), 3.80-3.60 (m, H-3"β, H-5"α), 3.62-3.72 (m, 2H, H-5'a), 3.33 (m, H-5"β), 2.28 (m, H-2"$_{ax}$β), 2.09, 2.07 (2s, C*H*$_3$CO), 2.08 (m, H-2"$_{eq}$β), 1.78 (ddd, *J* 2.9, 10.3, 12.3 Hz, H-2"$_{ax}$α), 1.59, 1.58, 1.37, 1.36 (4s, $C(CH_3)_2$), 1.19 (d, *J* 6.3 Hz, CH_3 (H-6"β), 1.15 (d, *J* 6.3 Hz, CH_3 (H-6"α), 0.85, (s, Si[C(C*H*$_3$)$_3$(CH$_3$)$_2$]α), 0.84 (s, Si[C(C*H*$_3$)$_3$(CH$_3$)$_2$]β), 0.06, 0.04 (2s, Si[C(CH$_3$)$_3$(C*H*$_3$)$_2$]α), 0.05, 0.03 (2s, Si[C(CH$_3$)$_3$(C*H*$_3$)$_2$]β). ^{13}C NMR data for α-anomer δ: 170.20, (CH$_3$*C*O), 163.25 (C-4), 150.18 (C-2), 141.42 (C-6), 114.88 (*C*(CH$_3$)$_2$), 102.92 (C-5), 98.23 (C-1"), 93.43 (C-1'), 85.73-66.60 (7C, C-2',3',4',5',3",4",5"), 39.04 (C-2"), 27.49, 25.74 ($C(CH_3)_2$), 25.60, 25.53 (Si[C(*C*H$_3$)$_3$(CH$_3$)$_2$]), 21.41 (*C*H$_3$CO), 18.03 (Si[*C*(CH$_3$)$_3$(CH$_3$)$_2$]), 17.89 (C-6"), -4.19, -4.56 (Si[C(CH$_3$)$_3$(*C*H$_3$)$_2$]);b ESI-LRMS: Calcd for $C_{26}H_{42}N_2O_{10}SiNa$ ([M+Na]$^+$): *m/z* 593, found: *m/z* 593.2.

2.1.2. 4-O-Acetyl-3-O-tert-butyldimethylsilyl-2,6-dideoxy-L-arabino-hexopyranosyl-(1→5)-3-N-benzoyl-2',3'-isopropylideneuridine (2)

Glycal **10** (86 mg, 0.30 mmol) and uridine derivative **15** (112 mg, 0.30 mmol) in CH_2Cl_2 were subjected to general procedure A (reaction time 2 h) yielding adduct **2** as an inseparable α,β-anomeric mixture (132 mg, 65% yield, colourless foam).
^{1}H NMR ($CDCl_3$): (α:β = 5:1) data for α-anomer δ 7.95-7.45 (m, 5H, Ph), 7.52 (d, 1H, *J* 8.1 Hz, H-6), 5.88 (d, 1H, *J* 8.1 Hz, H-5), 5.81 (d, 1H, *J* 2.4 Hz, H-1'), 4.88 (dd, 1H *J* 2.4, 6.3 Hz, H-2'), 4.85 (bd, 1H *J* 2.9 Hz, H-1"α), 4.74 (dd, 1H *J* 3.6, 6.3 Hz, H-3'), 4.66 (t, 1H *J* 9.3 Hz, H-4"), 4.52 (dd, *J* 1.9, 9.8 Hz, H-1"β), 4.36 (m, 1H H-4'), 3.98 (ddd, 1H *J* 5.1, 9.2, 11.2, H-3"), 3.87 (dd, 1H *J* 2.9, 11.2 Hz, H-5'b), 3.69 (m, 1H, H-5"), 3.52 (dd, 1H, *J* 5.8, 11.2 Hz, H-5'a), 2.12 (m, 1H, H-2"$_{eq}$), 2.09, (s, 3H, C*H*$_3$CO), 1.79 (m, 1H, H-2"$_{ax}$), 1.57, 1.35 (2s, $C(CH_3)_2$), 1.12 (d, 1H *J* 6.3 Hz, CH_3 (H-6"), 0.85, (s, Si[C(C*H*$_3$)$_3$(CH$_3$)$_2$]), 0.06, 0.04 (2s, Si[C(CH$_3$)$_3$(C*H*$_3$)$_2$]); ^{13}C NMR data for α-anomer δ 170.20, (CH$_3$*C*O), 168.29 (PhCO), 161.99 (C-4), 149.11 (C-2), 141.14 (C-6), 135.27 (Ph$_q$), 114.63 (*C*(CH$_3$)$_2$), 102.64 (C-5), 98.01 (C-1"), 94.06 (C-1'), 85.94-66.44 (7C, C-2',3',4',5',3",4",5"), 38.82 (C-2"), 27.22, 25.54 ($C(CH_3)_2$), 25.51, 25.36 (Si[C(*C*H$_3$)$_3$(CH$_3$)$_2$]), 21.18 (*C*H$_3$CO), 17.83 (Si[*C*(CH$_3$)$_3$(CH$_3$)$_2$]), 17.64 (C-6"), -4.19, -4.56 (Si[C(CH$_3$)$_3$(*C*H$_3$)$_2$]). ESI-LRMS: Calcd for $C_{33}H_{46}N_2O_{11}SiNa$ ([M+Na]$^+$): *m/z* 697, found: *m/z* 697.6.

*2.1.3. 3,4,6-Tri-O-benzyl-2-deoxy-D-glucopyranosyl-(1→5)-2,3-O-isopropylideneuridine (**3**)*

Glycal **11** (125 mg, 0.30 mmol) and uridine derivative **14** (85 mg, 0.30 mmol) in acetonitrile were subjected to general procedure A (reaction time 3 h) yielding adduct **3** as an inseparable α,β-anomeric mixture (164 mg, 78% yield, colourless foam).

^{1}H NMR ($CDCl_3$): (α:β = 4:1) data for α-anomer δ 8.90 (s, 1H, NH), 7.38-7.15 (m, 15 H, Ph), 7.36 (d, 1H, *J* 8.1 Hz, H-6), 5.79 (bs, 1H, H-1'), 5.64 (d, 1H, *J* 8.1 Hz, H-5), 4.97 (bd, 1H *J* 2.8 Hz, H-1"α), 4.90-4.46 ($3q_{AB}$, 6H, PhC*H*$_2$), 4.72-4.58 (m, 2H, H-2', H-3'),4.35 (m, 1H H-4'), 3.92-3.56 (m, 7H, H-3", H-4", H-5", H-6"a,b, H-5'a,b), 2.15 (m, 1H, H-2"$_{eq}$), 1.76 (m, 1H, H-2"$_{ax}$), 1.57, 1.35 (2s, 6H, $C(CH_3)_2$), ^{13}C NMR data for α-anomer δ 162.91 (C-4), 149.83 (C-2), 140.87 (C-6), 138.28, 138.15, 137.97 (Ph_q), 132.15-127.70 (Ph), 114.37 (*C*$(CH_3)_2$), 102.07 (C-5), 97.67 (C-1"), 93.20 (C-1'), 85.45-67.15 (11C, Ph*C*H$_2$ (3C), C-2',3',4',5', C-3",4",5",6"), 35.21 (C-2"), 27.26, 25.44 ($C(CH_3)_2$). ESI-LRMS: Calcd for $C_{39}H_{44}N_2O_{10}Na$ ($[M+Na]^+$): *m/z* 723, found: *m/z* 723.3.

*2.1.4. 3,4,6-Tri-O-benzyl-2-deoxy-D-glucopyranosyl-(1→5)-3-N-benzoyl-2,3-O-isopropylideneuridine (**4**)*

Glycal **11** (125 mg, 0.30 mmol) and uridine derivative **15** (112 mg, 0.30 mmol) in CH_2Cl_2 were subjected to general procedure A (reaction time 2 h) yielding adduct **4** as an inseparable α,β-anomeric mixture (141 mg, 58% yield, yellowish foam).

^{1}H NMR ($CDCl_3$): (α:β = 5:1) data for α-anomer δ 7.94-7.25 (m, 20 H, Ph), 7.49 (d, 1H, *J* 8.2 Hz, H-6), 5.78 (d, 1H, *J* 2.4 Hz, H-1'), 5.76 (d, 1H, *J* 8.2 Hz, H-5), 4.95 (bd, 1H *J* 2.9 Hz, H-1"α), 4.90-4.49 ($3q_{AB}$, 6H, PhC*H*$_2$), 4.76-4.66 (m, 2H, H-2', H-3'), 4.37 (m, 1H H-4'), 3.87 (ddd, 1H *J* 5.0, 9.0, 11.2, H-3"), 3.82-3.58 (m, 6H, H-4", H-5", H-6"a,b, H-5'a,b), 2.16 (m, 1H, H-2"$_{eq}$), 1.75 (m, 1H, H-2"$_{ax}$), 1.55, 1.33 (2s, 6H, $C(CH_3)_2$), ^{13}C NMR data for α-anomer δ 168.28 (PhCO), 161.89 (C-4), 149.04 (C-2), 140.66 (C-6), 138.32, 138.33, 138.18, 135.13 (Ph_q), 130.48-127.70 (Ph), 114.63 (*C*$(CH_3)_2$), 101.97 (C-5), 97.74 (C-1"), 93.97 (C-1'), 85.72-67.14 (11C, Ph*C*H$_2$ (3C), C-2',3',4',5', C-3",4",5",6"), 35.14 (C-2"), 27.23, 25.42 ($C(CH_3)_2$). ESI-LRMS: Calcd for $C_{33}H_{46}N_2O_{11}SiNa$ ($[M+Na]^+$): *m/z* 697, found: *m/z* 697.6. ESI-LRMS: Calcd for $C_{46}H_{48}N_2O_{11}Na$ ($[M+Na]^+$): *m/z* 827, found: *m/z* 827.3.

*2.1.5. 3,4,6-Tri-O-acetyl-2-deoxy-D-glucopyranosyl-(1→5)-2',3'-isopropylideneuridine (**6**)*

Glycal **13** (82 mg, 0.30 mmol) and uridine derivative **14** (85 mg, 0.30 mmol) in acetonitrile were subjected to general procedure A (reaction time 24 h) yielding adduct **6** as an inseparable α,β-anomeric mixture (77 mg, 46% yield, colourless foam).

^{1}H NMR ($CDCl_3$): (α:β = 3.5:1) data for α-anomer δ 9.35 (s, 1H, NH), 7.39 (d, 1H, *J* 8.1 Hz, H-6), 5.85 (d, 1H, *J* 2.3 Hz H-1'), 5.82 (d, 1H, *J* 8.1 Hz, H-5), 5.24 (ddd, 1H, *J* 5.2, 9.5, 11.6 Hz, H-3"), 5.03 (t, 1H, *J* 10.0 Hz, H-4"), 4.98 (bd, 1H *J* 2.6 Hz, H-1"α), 4.93 (dd, 1H, *J* 2.2, 6.3, Hz, H-2'), 4.84 (dd, 1H, *J* 4.1, 6.3 Hz, H-3'), 4.62 (dd, *J* 1.9, 9.0 Hz, H-1"β), 4.31 (m, 1H, H-4'), 4.28 (dd, 1H, *J* 4.6, 12.3 Hz, H-6b"), 4.05 (dd, 1H, *J* 2.4, 12.3 Hz, H-6a"), 3.92 (ddd, 1H, *J* 2.3, 4.6, 10.0 Hz, H-5"), 3.87 (dd, 1H, *J* 3.9, 11.1 Hz, H-5'b), 3.69 (dd, 1H, *J* 3.3, 11.1 Hz, H-5'a), 2.25 (ddd, 1H, *J* 1.2, 5.2, 13.0 Hz, H-2"$_{eq}$), 2.09, 2.06, 2.05 (3s, 9H, C*H*$_3$CO), 1.83 (ddd, 1H, *J* 3.5, 11.6, 13.0 Hz, H-2"$_{ax}$), 1.58, 1.38 (2s, 6H, $C(CH_3)_2$), ^{13}C NMR data for α-anomer δ 170.76, 170.37, 169.80 ($3CH_3$*C*O), 163.48 (C-4), 150.01 (C-2), 141.50 (C-6), 114.58 (*C*$(CH_3)_2$), 102.79 (C-5), 97.45 (C-1"), 92.96 (C-1'), 85.45-60.42 (8C, C-2',3',4',5', C-3",4",5",6"), 34.77 (C-2"), 27.29, 25.48 ($C(CH_3)_2$), 21.05, 20.95, 20.74 (3*C*H$_3$CO). ESI-LRMS: Calcd for $C_{24}H_{32}N_2O_{13}Na$ ($[M+Na]^+$): *m/z* 579, found: *m/z* 579.4.

2.1.6. *2-Deoxy-α-D-glucopyranosyl-(1→5)-3-N-benzyl-2',3'-isopropylideneuridine (**8**)*

Glycal **11** (125 mg, 0.30 mmol) and uridine derivative **16** (113 mg, 0.30 mmol) in CH_2Cl_2 were subjected to general procedure A (reaction time 2.5 h) yielding adduct, which was subsequently dissolved in a 1:10:2 mixture of cyclohexene : EtOH : THF (6 mL). The resulting solution was heated under reflux in the presence of $Pd(OH)_2/C$ (125 mg) for 1.5 h. After removal of the catalyst by filtration crude product was purified by column chromatography with $CHCl_3$/MeOH 20:1 solvent system to yield **8** (79 mg, 51% overall yield) as a colourless foam; $[\alpha]_D^{20}$ +27.3 (MeOH, c 0.5). ^{1}H NMR (CD_3OD): δ 7.78 (d, 1H, *J* 8.1 Hz, H-6), 7.38-7.20 (m, 5H, Ph), 5.86 (d, 1H, *J* 1.7 Hz, H-1'), 5.83 (d, 1H, *J* 8.1 Hz, H-5), 5.12, 5.04 (q_{AB}, 2H *J* 14.0 Hz, PhC*H*$_2$), 4.92 (bd, 1H, *J* 2.9 Hz, H-1"α), 4.87-4.81 (m, 2H, H-2', H-3'), 4.43 (m, 1H H-4'), 3.91-3.44 (m, 6H, H-3", H-5", H-6"a,b, H-5'a,b), 3.22 (t, 1H, *J* 9.5 Hz, H-4"), 1.98 (m, 1H, H-2"$_{eq}$), 1.61 (m, 1H, H-2"$_{ax}$), 1.54, 1.35 (2s, 6H, $C(CH_3)_2$), ^{13}C NMR δ 164.91 (C-4), 152.29 (C-2), 141.50 (C-6), 138.14 (Ph_q), 129.49, 129.27, 128.55 (Ph), 114.94 (*C*$(CH_3)_2$), 101.77 (C-5), 98.87 (C-1"), 95.47 (C-1'), 87.29-62.79 (8C, C-2',3',4',5', C-3",4",5",6"), 45.14 (Ph*C*H_2N), 38.63 (C-2"), 27.57, 25.55 ($C(CH_3)_2$). ESI-LRMS: Calcd for $C_{25}H_{32}N_2O_{10}Na$ ($[M+Na]^+$): *m/z* 543, found: *m/z* 543.9.

3. *Determination of the chromatographic lipophilicity index R_{Mw} by RP-TLC chromatography*

The TLC experiments were performed on Silica gel 60 RP-18 F_{254} plates (Merck), constituting the non-polar stationary phase. Methanol/water solvent systems were applied as mobile phases. Amounts of methanol as an organic modifier were in range 60-85% (*v/v*) in 5% increments.
Solutions of the examined compounds were prepared in methanol in concentrations of about 1 mg/ml. An elongated spot of solution of each compound (about 5 μL) was deposited on a plate (size 100 mm x 100 mm), the plate was developed in a classical flat-bottomed glass chamber with an appropriate eluent. Then the solvent front was marked, plates were dried and visualized in UV light at λ = 254 nm. The center of each spot was determined precisely and marked. The R_f values were averaged from two to three determinations and converted into R_M values via the relationship $R_M=\log[(1/R_f)-1]$. Using methanol as the organic modifier, R_M values were linearly correlated to the organic modifier percentage in the mobile phase as described by:

$$R_M = R_{Mw} + sC \quad (1)$$

where R_{Mw} is the chromatographic lipophilicity index extrapolated to 100% water as the mobile phase, C is concentration of methanol (%, *v/v*) and s is a constant for a given TLC system.
